# Development of a machine learning-based model to predict prognosis of alpha-fetoprotein-positive hepatocellular carcinoma

**DOI:** 10.1186/s12967-024-05203-w

**Published:** 2024-05-13

**Authors:** Bingtian Dong, Hua Zhang, Yayang Duan, Senbang Yao, Yongjian Chen, Chaoxue Zhang

**Affiliations:** 1 Department of Ultrasound, the First Affiliated Hospital of Anhui Medical University, Hefei, China; 2 Department of Ultrasound, Affiliated Hangzhou First People’s Hospital, School of Medicine, Westlake University, Hangzhou, China; 3 Department of Oncology, The Second Affiliated Hospital of Anhui Medical University, Hefei, Anhui China; 4 Department of Oncology, Anhui Medical University, Hefei, Anhui China; 5 Department of Ultrasound, The Second Affiliated Hospital of Fujian Medical University, Quanzhou, China

**Keywords:** Hepatocellular carcinoma, Predictive analytics, Survival, Machine learning, XGBoost algorithm

## Abstract

**Background:**

Patients with alpha-fetoprotein (AFP)-positive hepatocellular carcinoma (HCC) have aggressive biological behavior and poor prognosis. Therefore, survival time is one of the greatest concerns for patients with AFP-positive HCC. This study aimed to demonstrate the utilization of six machine learning (ML)-based prognostic models to predict overall survival of patients with AFP-positive HCC.

**Methods:**

Data on patients with AFP-positive HCC were extracted from the Surveillance, Epidemiology, and End Results database. Six ML algorithms (extreme gradient boosting [XGBoost], logistic regression [LR], support vector machine [SVM], random forest [RF], K-nearest neighbor [KNN], and decision tree [ID3]) were used to develop the prognostic models of patients with AFP-positive HCC at one year, three years, and five years. Area under the receiver operating characteristic curve (AUC), confusion matrix, calibration curves, and decision curve analysis (DCA) were used to evaluate the model.

**Results:**

A total of 2,038 patients with AFP-positive HCC were included for analysis. The 1-, 3-, and 5-year overall survival rates were 60.7%, 28.9%, and 14.3%, respectively. Seventeen features regarding demographics and clinicopathology were included in six ML algorithms to generate a prognostic model. The XGBoost model showed the best performance in predicting survival at 1-year (train set: AUC = 0.771; test set: AUC = 0.782), 3-year (train set: AUC = 0.763; test set: AUC = 0.749) and 5-year (train set: AUC = 0.807; test set: AUC = 0.740). Furthermore, for 1-, 3-, and 5-year survival prediction, the accuracy in the training and test sets was 0.709 and 0.726, 0.721 and 0.726, and 0.778 and 0.784 for the XGBoost model, respectively. Calibration curves and DCA exhibited good predictive performance as well.

**Conclusions:**

The XGBoost model exhibited good predictive performance, which may provide physicians with an effective tool for early medical intervention and improve the survival of patients.

**Supplementary Information:**

The online version contains supplementary material available at 10.1186/s12967-024-05203-w.

## Introduction

Hepatocellular carcinoma (HCC) is the most common form of liver cancer, accounting for approximately 75‒85% of cases [1, 2]. It is a highly fatal cancer and a major cause of cancer-related death worldwide, leading to more than 700,000 deaths each year [3].

Alpha-fetoprotein (AFP) is often expressed at high levels in HCC, and approximately 75% of patients with HCC were AFP positive [4, 5]. Compared to patients with AFP-negative HCC, patients with AFP-positive HCC were associated with worse biological behavior and inferior survival [4, 6]. Patients with AFP-positive HCC were more likely to present with higher clinical stage, TNM classification, fibrosis scores, and a more vessel invasion [4, 7, 8]. A recent study showed that regardless of surgical or adjuvant therapy, the median overall survival time of patients with AFP-positive HCC was much lower than those of patients with AFP-negative HCC (13 months vs. 48 months) [4]. Therefore, it is imperative to create prognostic prediction models for patients with AFP-positive HCC, thereby contributing to accurately answer their concerns about survival and helping to implement individualized management.

Machine learning, a new type of artificial intelligence (AI), has recently become a topic of paramount importance, providing methods, techniques, and tools for the analysis of data generated by the biological sciences [9–11]. It can learn from examples to make patient-level survival predictions and establish clinical AI prognostic models with significantly improved accuracy [9, 12]. Extreme gradient boosting (XGBoost) is a newer ensemble-learning algorithm, which can be applied to adjust the errors generated by existing models [13, 14]. XGBoost has been used for effective survival prediction of cancer patients [14–17]. However, it has rarely been applied for the prediction of prognosis for patients with AFP-positive HCC.

In this study, we implemented six machine learning algorithms including XGBoost, logistic regression (LR), support vector machine (SVM), random forest (RF), K-nearest neighbor (KNN), and decision tree (ID3) to predict 1-, 3- and 5-year survival of patients with AFP-positive HCC, using data retrieved from the Surveillance, Epidemiology, and End Results (SEER) database. The present study contributes to developing machine learning-based models to provide insight into the prognosis of patients with AFP-positive HCC.

## Methods

### Data source and patient selection

**Fig. 1:**
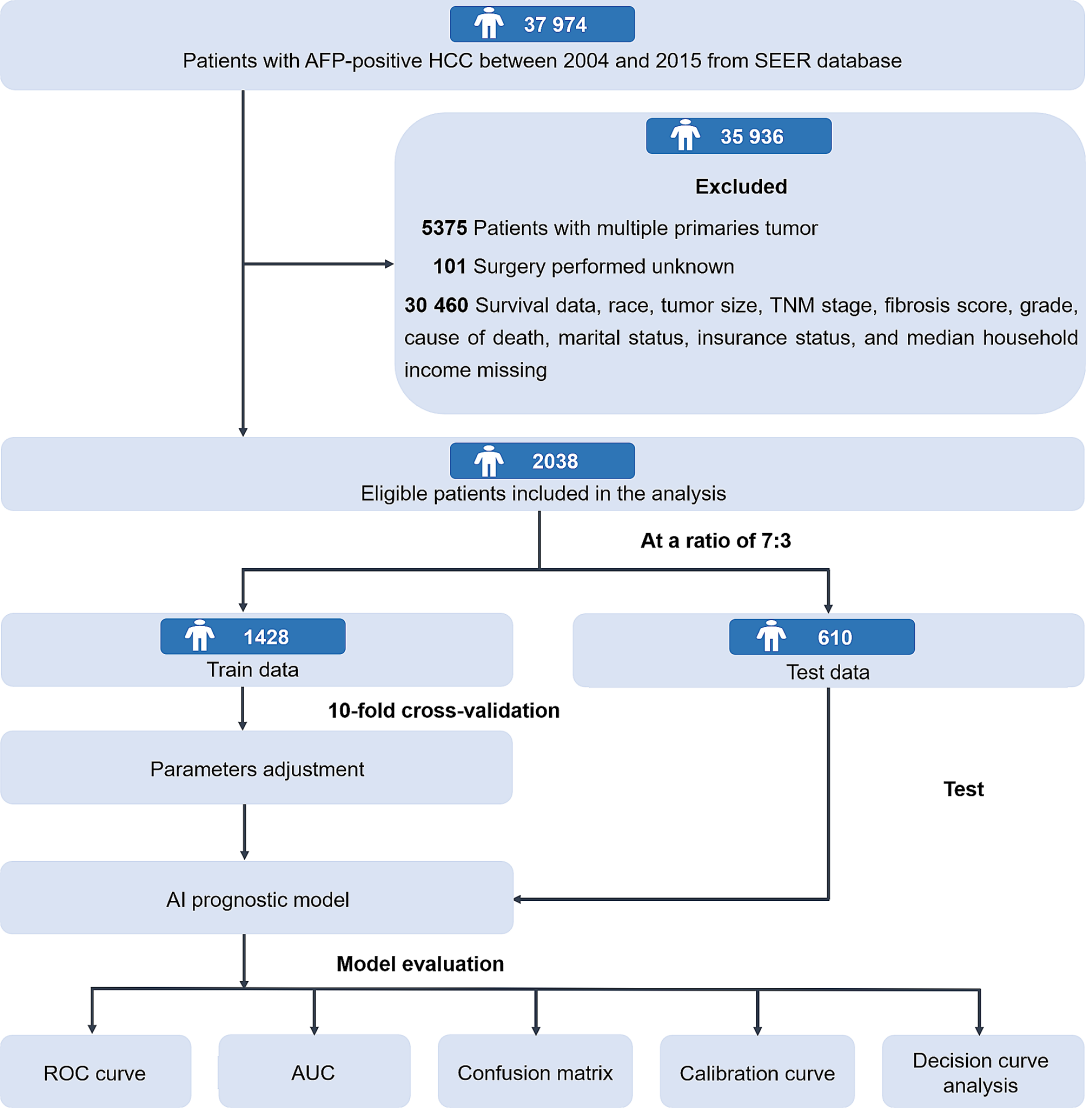
Flowchart of study design and patient selection. *AFP* alpha-fetoprotein; *HCC* hepatocellular carcinoma; *SEER* Surveillance, Epidemiology, and End Results; *TNM* tumor lymph node metastasis; *ROC curve* receiver operating characteristic curve; *AUC* area under the curve

Data on patients with AFP-positive HCC were extracted from the SEER database, which is an important population-based program of the National Cancer Institute and covers approximately 30% of the United States population [18]. According to the International Classification of Diseases for Oncology, Third Edition (ICD-O-3), the inclusion primary site code was C22.0 and the histological codes were 8170/3‒8175/3. Patients diagnosed between 2004 and 2015 were collected. The following cases were excluded: (1) patients with AFP-negative HCC patients; (2) patients with multiple primary tumors; (3) incomplete information including tumor size, race, survival data, AFP, fibrosis score, grade, cause of death, marital status, insurance status, and median household income; (4) unknown TNM stage; and (5) unknown whether surgery was performed. Finally, 2,038 eligible patients with AFP-positive HCC were included and further analyzed in this study. Figure 1 presents the flowchart of study design and patient selection.

### Study variables

The following factors were included as explanatory variables: race, sex, age at diagnosis, histological grade, tumor size, TNM stage [American Joint Committee on Cancer (AJCC) 7th version], SEER stage, fibrosis score, marital status, insurance status, median household income, and treatment strategy (surgery, radiotherapy, and chemotherapy). The outcome variables were survival months and overall survival.

### XGBoost model

XGBoost is a newer ensemble-learning algorithm, which was officially published in 2016 [13, 14]. It is more novel and complex compared to traditional machine learning algorithms [19]. The basic concepts of each machine learning algorithm are presented in Supplementary Text 1. In this study, the model was built on the training set by 10-fold cross-validation, in order to ensure the stability of the model. We tested and adjusted the model repeatedly and finally determined the key hyperparameters. In addition, a test set was devoted to further validate the model. Here, we aimed to develop a machine learning-based model to predict the overall survival of patients with AFP-positive HCC at 1-, 3-, and 5-year.

### Statistical analysis

In terms of basic characteristics, categorical variables were presented as number (*n*) and percentage (%). Chi-square test was used to compare differences between training and test sets. Normally distributed continuous variables were expressed as mean ± standard deviation, and non-normally distributed continuous variables were illustrated as median (range). When appropriate, *t* test or Mann-Whitney *U* test was used. Age, tumor size, and median household income were presented as continuous variables.

In this study, six machine learning algorithms (XGBoost, LR, SVM, RF, KNN, and ID3) were used to develop the prognostic models for patients with AFP-positive HCC. We evaluated the predictive performance of six machine learning-based prognostic models using the receiver operating characteristic (ROC) analysis and confusion matrix. Area under the ROC curve (AUC) was calculated to evaluate the model, using the ROC curve analysis. Accuracy was also calculated, which is one of the primary assessment parameters in the confusion matrix [15]. In addition, calibration curves and decision curve analyses (DCA) were also performed. All statistical analyses were performed with SPSS version 26 and Python version 3.6 (Python Software Foundation). A *P* value < 0.05 was considered statistically significant.

## Results

### Patient characteristics

We obtained the information on 2,038 eligible patients with AFP-positive HCC from the SEER program. The 1-, 3-, and 5-year overall survival rates of patients with AFP-positive HCC were 60.7%, 28.9%, and 14.3%, respectively. The baseline characteristics of the training and test sets are shown in Table 1 and summarized below. There was no difference in baseline data (except for marital status and median household income) between the training and test sets.

**Table 1:** Baseline characteristics of AFP-positive HCC patients

Characteristics	Total (*n* = 2,038) n (%)	Training set (*n* = 1,428) *n* (%)	Test set (*n* = 610) *n* (%)	χ^2^/t/Z	*P* value
Age (mean ± SD), years	61.07 ± 10.11	61.04 ± 10.29	61.12 ± 9.68	-0.151	0.880
Sex				2.746	0.098
Male	1,555 (76.3)	1,075 (75.3)	480 (78.7)		
Female	483 (23.7)	353 (24.7)	130 (21.3)		
Race				3.162	0.206
White	1,293 (63.4)	892 (62.5)	401 (65.8)		
Black	290 (14.2)	202 (14.1)	88 (14.4)		
Others	455 (22.3)	334 (23.4)	121 (19.8)		
Marital status				5.027	0.025
Married	1,179 (57.9)	849 (59.5)	330 (54.1)		
Others	859 (42.1)	579 (40.5)	280 (45.9)		
Grade				2.743	0.433
I	541 (26.5)	368 (25.8)	173 (28.4)		
II	1,022 (50.2)	726 (50.8)	296 (48.5)		
III	446 (21.9)	311 (21.8)	135 (22.1)		
IV	29 (1.4)	23 (1.6)	6 (1.0)		
Tumor size (mm)					
Median (range)	40.00 (4-850)	40.00 (4-850)	41.00 (6-461)	-1.052	0.293
AJCC stage				3.958	0.266
I	807 (39.6)	578 (40.5)	229 (37.5)		
II	647 (31.8)	459 (32.1)	188 (30.8)		
III	437 (21.4)	294 (20.6)	143 (23.4)		
IV	147 (7.2)	97 (6.8)	50 (8.2)		
AJCC T stage				4.942	0.176
T1	843 (41.4)	604 (42.3)	239 (39.2)		
T2	695 (34.1)	490 (34.3)	205 (33.6)		
T3	437 (21.4)	288 (20.2)	149 (24.4)		
T4	63 (3.1)	46 (3.2)	17 (2.8)		
AJCC N stage				0.131	0.718
N0	1,930 (94.7)	1,354 (94.8)	576 (94.4)		
N1	108 (5.3)	74 (5.2)	34 (5.6)		
AJCC M stage				1.259	0.262
M0	1,891 (92.8)	1,331 (93.2)	560 (91.8)		
M1	147 (7.2)	97 (6.8)	50 (8.2)		
Surgery				0.924	0.336
Yes	1,202 (59.0)	852 (59.7)	350 (57.4)		
No	836 (41.0)	576 (40.3)	260 (42.6)		
Radiotherapy				0.275	0.600
Yes	136 (6.7)	98 (6.9)	38 (6.2)		
No	1,902 (93.3)	1,330 (93.1)	572 (93.8)		
Chemotherapy				0.998	0.318
Yes	848 (41.6)	584 (40.9)	264 (43.3)		
No/unknown	1,190 (58.4)	844 (59.1)	346 (56.7)		
SEER stage				1.086	0.581
Localized	1,271 (62.4)	901 (63.1)	370 (60.7)		
Regional	616 (30.2)	423 (29.6)	193 (31.6)		
Distant	151 (7.4)	104 (7.3)	47 (7.7)		
Fibrosis score				1.646	0.199
0–4	530 (26.0)	383 (26.8)	147 (24.1)		
5–6	1,508 (74.0)	1,045 (73.2)	463 (75.9)		
Insurance status				2.415	0.299
Any medicaid	481 (23.6)	342 (23.9)	139 (22.8)		
Insured	1,509 (74.0)	1,048 (73.4)	461 (75.6)		
Uninsured	48 (2.4)	38 (2.7)	10 (1.6)		
Median household income^a^					
Median (range)	3,904 (1597–6275)	3,904 (1802–6275)	3,904 (1597–6275)	-2.305	0.021

^a^Median household income (in tens) in U.S. dollars

*AFP* alpha-fetoprotein; *AJCC* American Joint Committee on Cancer; *HCC* Hepatocellular Carcinoma; *SD* standard deviation; *SEER* Surveillance, Epidemiology, and End Results

Of these patients, 76.3% were male, and 63.4% were white. The average age was 61.07 years. Patients with grade III or IV tumors accounted for 23.3%. In terms of marital status, about 57.9% of patients were married. There were 1,509 (74.0%) patients who were insured. The majority of patients (74.0%) had a high fibrosis score (fibrosis score 5–6, i.e., severe fibrosis or cirrhosis). Regarding tumor size, tumors with ≤ 3 cm, 3–5 cm, and ≥ 5 cm accounted for 33.3%, 27.2%, and 39.5% of patients, respectively. In the treatment field, across the entire study population, more than half of the patients received surgical treatment, accounting for approximately 59.0%, followed by 41.6% with chemotherapy, while only 6.7% received radiotherapy.

### Feature predictor selection

**Fig. 2:**
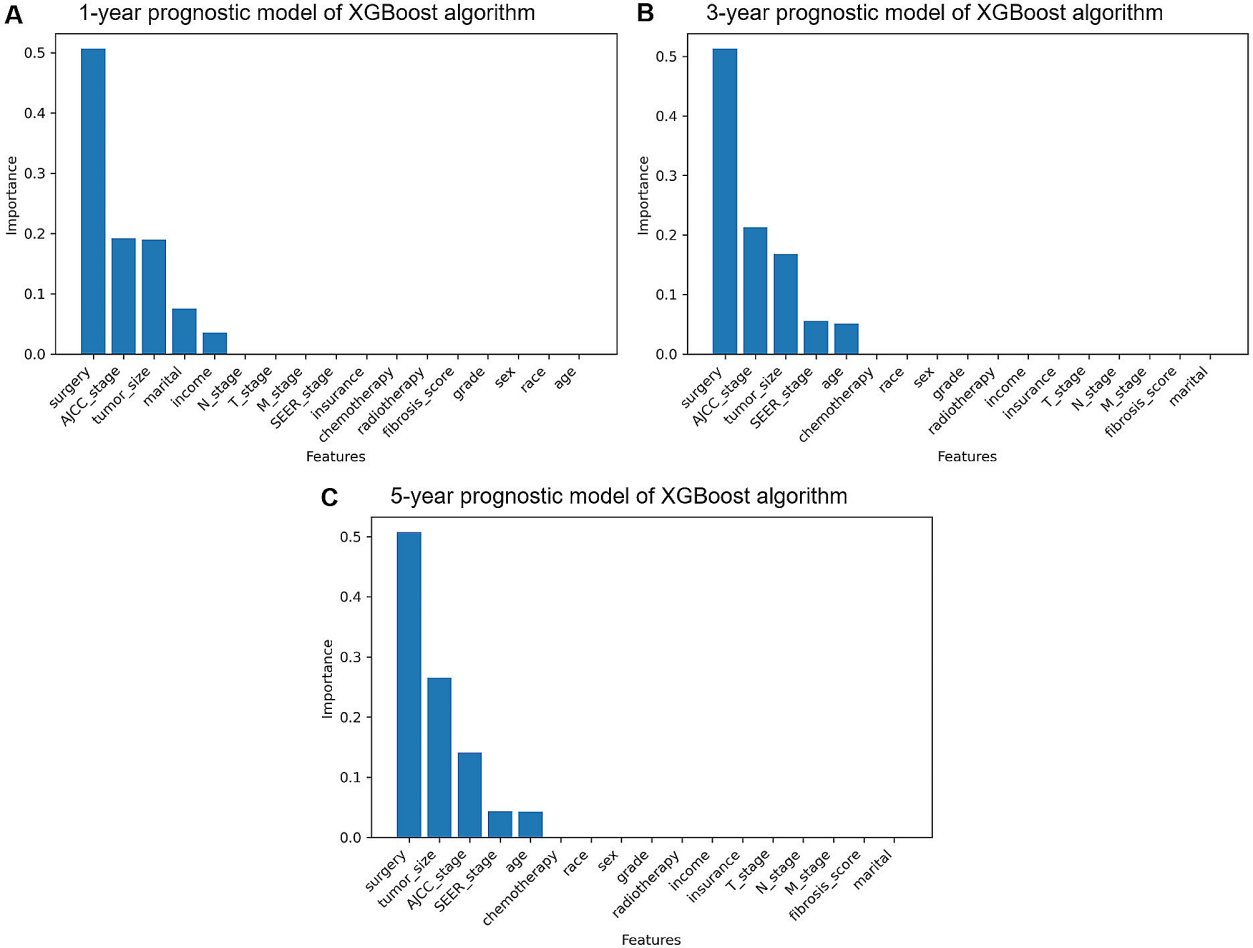
The importance of each feature in the XGBoost prognostic model. **A** The importance of each feature in the 1- year prognostic model; **B** the importance of each feature in the 3-year prognostic model; **C** the importance of each feature in the 5-year prognostic model. *XGBoost* extreme gradient boosting

The importance of each feature in the XGBoost prognostic model is illustrated in Fig. 2. The findings revealed that for the 1-year prognostic model, the top five variables affecting prognosis were surgery, AJCC stage, tumor size, marital status, and median household income, while surgery, AJCC stage, tumor size, SEER stage, and age were the top five variables for 3- and 5-year prognostic models. Among them, surgery was the most important variable for 1-, 3- and 5-year prognostic models of XGBoost.

### Construction of AI prognostic model

The total cases were randomly divided into a training set (*n* = 1,428) and a test set (*n* = 610) at a ratio of 7:3, for the construction and verification of AI prognostic models, respectively. In the training set, we used ten-fold cross-validation for iterative testing and tuning, and tested and adjusted the model repeatedly. The key hyperparameters were finally confirmed. The main parameters of the XGBoost model are summarized as follows: Colsample_bytree = 0.8, Gamma = 0, Learning_rate = 0.1, Max_depth = 1, Min_child_weight = 1, and Subsample = 1.

### Evaluating predictive models for estimating the prognosis of patients with AFP-positive HCC

**Fig. 3:**
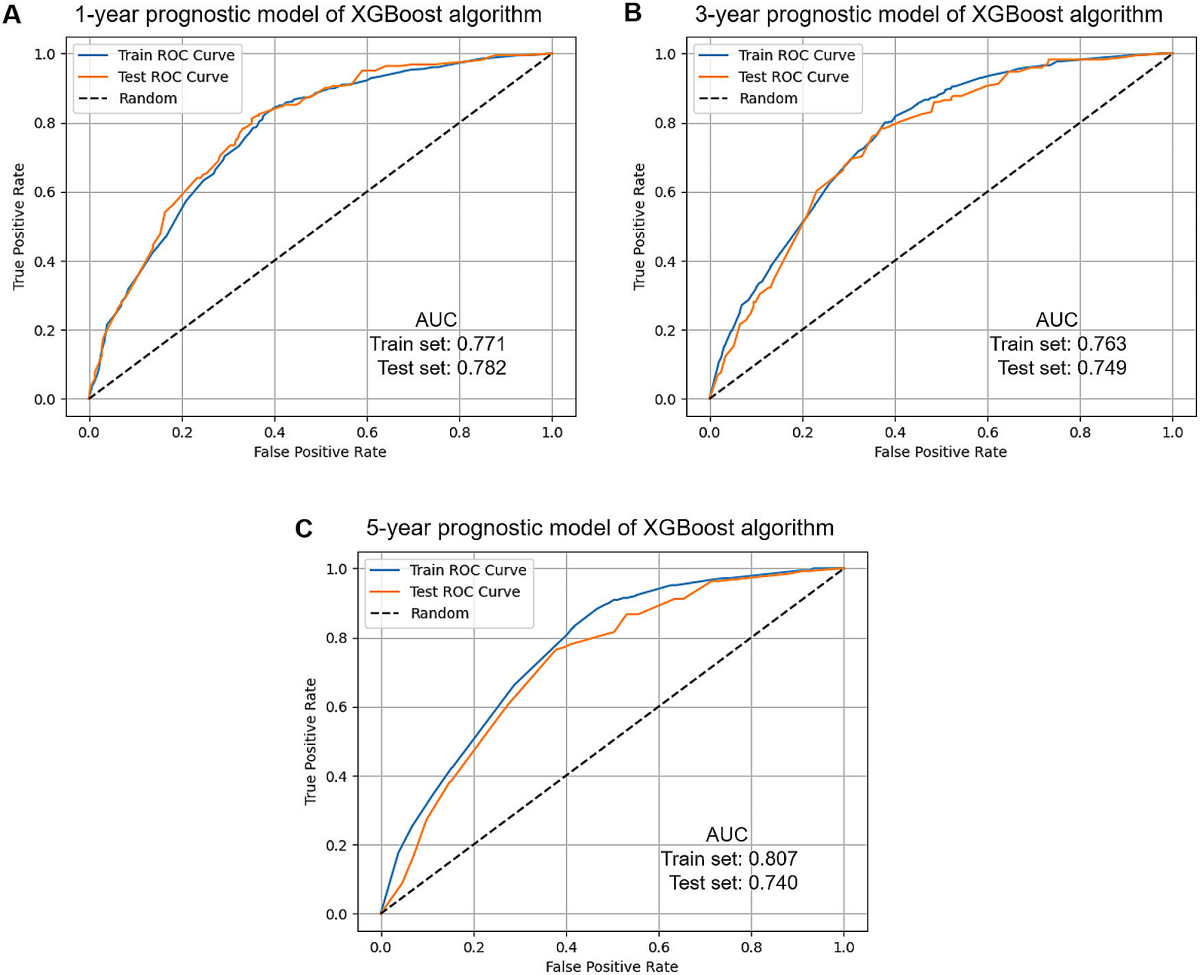
XGBoost model evaluation. **A** ROC curve for the 1-year prognostic model in the training and test sets; **B** ROC curve for the 3-year prognostic model in the training and test sets; **C** ROC curve for the 5-year prognostic model in the training and test sets. *XGBoost* extreme gradient boosting; *ROC* receiver operating characteristic curve; *AUC* area under the curve

Using ROC curve analysis, we calculated the corresponding AUCs for the training and test sets. The XGBoost model performed well in predicting survival of patients with AFP-positive HCC at 1-year (train set: AUC = 0.771; test set: AUC = 0.782), 3-year (train set: AUC = 0.763; test set: AUC = 0.749) and 5-year (train set: AUC = 0.807; test set: AUC = 0.740) (Fig. 3).

In the ROC curve analysis, the 1-year AUC values of LR, SVM, RF, KNN, and ID3 were 0.758, 0.703, 0.761, 0.746, and 0.762, respectively, in the training set, corresponding to 0.750, 0.734, 0.779, 0.631, and 0.750 in the test set (Table 2). In the 3-year prognostic model, the AUC values of LR, SVM, RF, KNN, and ID3 were 0.756, 0.687, 0.760, 0.744, and 0.752, respectively, in the training set, corresponding to 0.740, 0.739, 0.753, 0.607, and 0.718 in the test set. In the 5-year prognostic model, the AUC values of LR, SVM, RF, KNN, and ID3 were 0.753, 0.686, 0.754, 0.786, and 0.748, respectively, in the training set, corresponding to 0.708, 0.715, 0.718, 0.586, and 0.699 in the test set. Compared to the five machine learning algorithms, the XGBoost model performed the best.

**Table 2:** Performance of prognostic models built by machine learning algorithms in the training and test sets (area under the ROC curve)

	1-year survival	3-year survival	5-year survival
Training set			
XGBoost	0.771	0.763	0.807
LR	0.758	0.756	0.753
SVM	0.703	0.687	0.686
RF	0.761	0.760	0.754
KNN	0.746	0.744	0.786
ID3	0.762	0.752	0.748
Test set			
XGBoost	0.782	0.749	0.740
LR	0.750	0.740	0.708
SVM	0.734	0.739	0.715
RF	0.779	0.753	0.718
KNN	0.631	0.607	0.586
ID3	0.750	0.718	0.699

*ROC* receiver operating characteristic curve; *XGBoost* extreme gradient boosting; *LR* logistic regression; *SVM* support vector machine; *RF* random forest; *KNN* K-nearest neighbor; *ID3* decision tree

Furthermore, we evaluated the accuracy of the XGBoost model by constructing a confusion matrix (Supplementary Fig. 1). For 1-, 3-, and 5-year survival prediction, the accuracy in the training and test sets was 0.709 and 0.726, 0.721 and 0.726, and 0.778 and 0.784, respectively. Supplementary Table 1 shows the accuracy of each model in predicting 1-, 3-, and 5-year survival in the training and test sets.

**Fig. 4:**
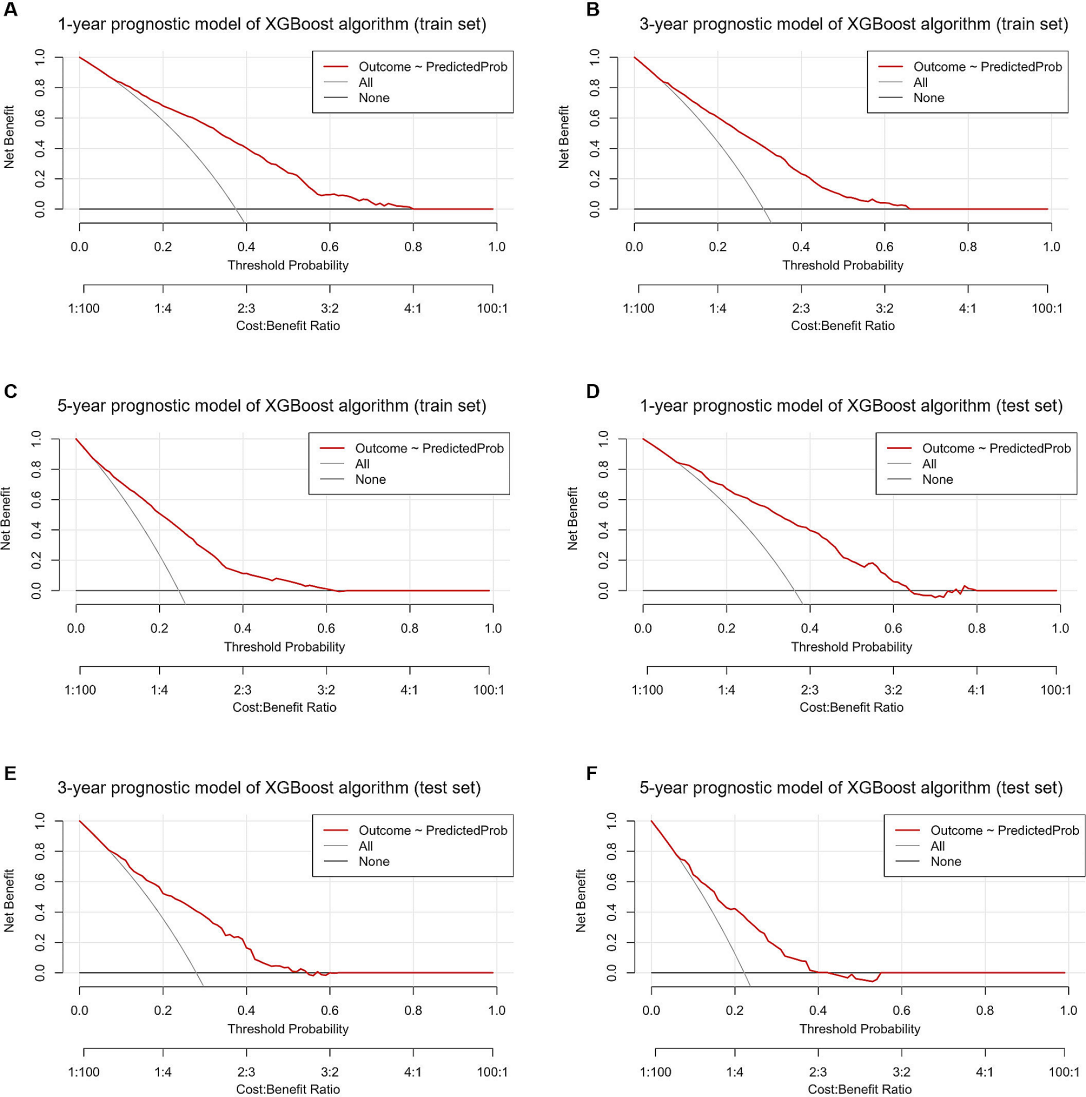
Decision curve analysis curves of the XGBoost model in the training and test sets. Decision curve analysis curves for **A** 1-year, **B** 3-year, and **C** 5-year prognostic models in the training set; and **D** 1-year, **E** 3-year, and **F** 5-year prognostic models in the test set. *XGBoost* extreme gradient boosting

The XGBoost model-related calibration curves displayed good consistency in the probability of 1-, 3-, and 5-year survival between the actual observation and the model prediction in the training (Supplementary Fig. 2A, B and C; respectively) and test (Supplementary Fig. 2D, E and F; respectively) sets. Meanwhile, the DCA curves of 1-, 3-, and 5-year survival in the training (Fig. 4A, B and C; respectively) and test (Fig. 4D, E and F; respectively) sets also demonstrated good clinical utility, showing preferable positive net benefit.

## Discussion

Patients with AFP-positive HCC have aggressive biological behavior and poor prognosis, therefore, survival time is one of the greatest concerns [4]. In current clinic practice, however, there is a lack of reliable predictive models. Accurate and powerful models are thus clearly needed. In this study, we developed six machine learning-based prognostic models for AFP-positive HCC to comprehensively analyze survival data. The 1-, 3-, and 5-year overall survival rates of AFP-positive HCC patients were 60.7%, 28.9%, and 14.3%, respectively.

To our knowledge, the current study is the first investigation to create AI prognostic models for patients with AFP-positive HCC. The XGBoost model showed good prediction accuracy, and the AUCs of the ROC curves in 1-, 3- and 5-year overall survival were 0.771, 0.763, and 0.807, respectively, in the training set, corresponding to 0.782, 0.749, and 0.740 in the test set. Compared to the five machine learning algorithms including LR, SVM, RF, KNN, and ID3, our results revealed that the XGBoost model performed best. It holds promise for early medical intervention and improving the survival of patients.

In recent years, machine learning-based AI models attracted increasing attention in clinical practice [14, 20, 21]. Especially, AI-based technologies have made a significant contribution to the field of cancer research [21]. Recent studies have examined the use of the XGBoost model in predicting the survival of cancer patients, and verified that this model is of better prediction ability in various types of cancer. In a recent study, Xu et al. [14] reported that the XGBoost model exhibited a better performance than the AJCC staging system in predict postoperative survival in elderly intrahepatic cholangiocarcinoma patients, with the AUCs of more than 0.7 both in the training and test sets. Li et al. [15] found that the XGBoost model behaved efficiently and successfully in predict the survival of patients with breast cancer brain metastases, with an AUC of 0.8 or above (test data). In addition, Zhong et al. [16] applied the XGBoost algorithm to create a prognostic model for patients with breast cancer with bone metastasis and showed AUC values of 0.88 and 0.80 in the training and test sets. Consistent with the previous studies [14–16], our present study also revealed that the XGBoost model showed good performance in prognostic survival prediction models, showing AUCs greater than 0.7 and even the 5-year AUC value over 0.8 (training data). Generally, an AUC ≥ 0.7 indicates that the model has an adequate predictive ability [22]. This suggests that XGBoost is an efficient machine learning classifier.

Notably, in this study, a total of 17 features in the basic characteristics of patients with AFP-positive HCC were considered in the survival prediction, which could be helpful in providing a comprehensive and accurate prediction. Our findings revealed that surgery, AJCC stage, tumor size, marital status, median household income, SEER stage, and age were relatively important variables affecting prognosis. Among them, surgery was the most important one. This is consistent with previous results. Several recent studies showed that surgery was an independent prognostic factor for patients with HCC [23–26]. Currently, surgical resection is still considered to be the gold standard treatment for HCC [27]. This result suggested the importance of surgical treatment in AFP-positive HCC, which is a favorable conclusion for both clinicians and patients. Consistently, AJCC stage, tumor size, and age were related to the survival of HCC patients [23, 25]. Previous studies have shown that patients with HCC with a tumor diameter ≤ 3 cm was low malignant potential and had better survival after treatment [28, 29]. Of note, age, tumor size, and median household income were presented as continuous variables rather than categorical variables. This implies that individualized survival prediction could be made for a particular patient, as opposed to a collective prediction for a group of patients, thus highlighting the concept of personalized prognosis prediction. In this study, marital status and median household income, two socio‑economic factors, were also identified as important predictors for survival in patients with AFP-positive HCC. Psychological and economic support from spouses may help to improve survival in married patients [30].

This study has its unique aspects. This is the first study to create AI prognostic models for patients with AFP-positive HCC. We implemented six machine learning algorithms and used ten-fold cross-validation for iterative testing and tuning, and tested and adjusted the model repeatedly. Moreover, based on different machine learning algorithms, we comprehensively analyzed 17 demographic/clinicopathological features, thus helping to provide an accurate prediction. Nonetheless, the present study has some potential limitations. First, this is a retrospective study. Second, we obtained the information on patients with AFP-positive HCC from the SEER database and, therefore, representativeness for other populations may be limited. Third, some other important information, such as concrete values of AFP, vascular invasion, etiology of HCC, and serum biochemical parameters, was not available in the SEER program. The model may miss some important features and lead to results bias. For example, previous studies revealed that microvascular invasion was an important and independent prognostic factor for patients with HCC [31, 32]. Finally, the AI prognostic models we created were internally validated, and despite their promising predictive performance, external validation using prospective studies is required to assess their applicability.

## Conclusions

In conclusion, our study developed six novel machine learning-based prognostic models for the survival of patients with AFP-positive HCC. The XGBoost model exhibited good predictive performance, which may provide physicians with an effective tool for early medical intervention and improve the survival of patients.

### Electronic supplementary material

Below is the link to the electronic supplementary material.

Supplementary Material 1

## Data Availability

Publicly available datasets were analyzed in this study. This data can be found here: https://seer.cancer.gov/.

## Abbreviations

HCC: Hepatocellular carcinoma
AFP: Alpha-fetoprotein
AI: Artificial intelligence
XGBoost: Extreme gradient boosting
SEER: Surveillance, Epidemiology, and End Results
ICD-O-3: International Classification of Diseases for Oncology, Third Edition
AJCC: American Joint Committee on Cancer
LR: Logistic regression
SVM: Support vector machine
RF: Random forest
KNN: K-nearest neighbor
ID3: Decision tree
ROC: Receiver operating characteristic
AUC: Area under the receiver operating characteristic curve
DCA: Decision curve analyses
