# Supplementary material for: Development of a machine learning-based model to predict prognosis of alpha-fetoprotein-positive hepatocellular carcinoma
Source: J Transl Med. 2024 May 13;22:455. doi: 10.1186/s12967-024-05203-w (PMC11092049; doi:10.1186/s12967-024-05203-w)
Supplement: Supplementary file 1 — Supplementary Material 1 [file 12967_2024_5203_MOESM1_ESM.docx]

**Supplementary Text 1** Overview of machine learning models

In this study, we implemented six machine learning algorithms including extreme gradient boosting (XGBoost), logistic regression (LR), support vector machine (SVM), random forest (RF), K-nearest neighbor (KNN), and decision tree (ID3) to predict 1-, 3- and 5-year survival of patients with AFP-positive HCC.

XGBoost is a newer ensemble-learning algorithm, which was officially published in 2016 [1,2]. It is an optimized distributed gradient enhancement library consisting of multiple classification and regression trees, which is more novel and complex compared to traditional machine learning algorithms [3,4]. LR is a representative linear classifier, which assesses the relationship between a dependent variable and one or more independent variables [4,5]. SVM is a generalized linear classifier for binary classification of data according to supervised learning, and its goal is to establish the optimal hyperplane to distinguish positive and negative samples [4]. RF is an integrated learning method that performs classification or regression tasks by combining multiple decision tree models. RF has many advantages, such as being able to process high-dimensional data. KNN is an instance-based learning algorithm. It is easy to understand and suitable for multi-class classification and nonlinear decision boundary problems [6]. ID3 is a common decision tree algorithm, which makes decisions based on a tree structure. The core of ID3 is to select the features according to the information gain, and then recursively construct the decision tree.

**References**

1. Chen T, Guestrin C. XGBoost: A Scalable Tree Boosting System. 2016.
2. Xu Q, Lu X. Development and validation of an XGBoost model to predict 5-year survival in elderly patients with intrahepatic cholangiocarcinoma after surgery: a SEER-based study. J Gastrointest Oncol. 2022;13(6):3290-9.
3. Jiang J, Pan H, Li M, Qian B, Lin X, Fan S. Predictive model for the 5-year survival status of osteosarcoma patients based on the SEER database and XGBoost algorithm. Sci Rep. 2021;11(1):5542.
4. Fan R, Yu N, Li G, Arshad T, Liu WY, Wong GL, et al. Machine-learning model comprising five clinical indices and liver stiffness measurement can accurately identify MASLD-related liver fibrosis. Liver Int. 2024;44(3):749-59.
5. Jiang X, Zhang Y, Li Y, Zhang B. Forecast and analysis of aircraft passenger satisfaction based on RF-RFE-LR model. Sci Rep. 2022;12(1):11174.
6. Rui F, Yeo YH, Xu L, Zheng Q, Xu X, Ni W, et al. Development of a machine learning-based model to predict hepatic inflammation in chronic hepatitis B patients with concurrent hepatic steatosis: a cohort study. EClinicalMedicine. 2024;68:102419.

**
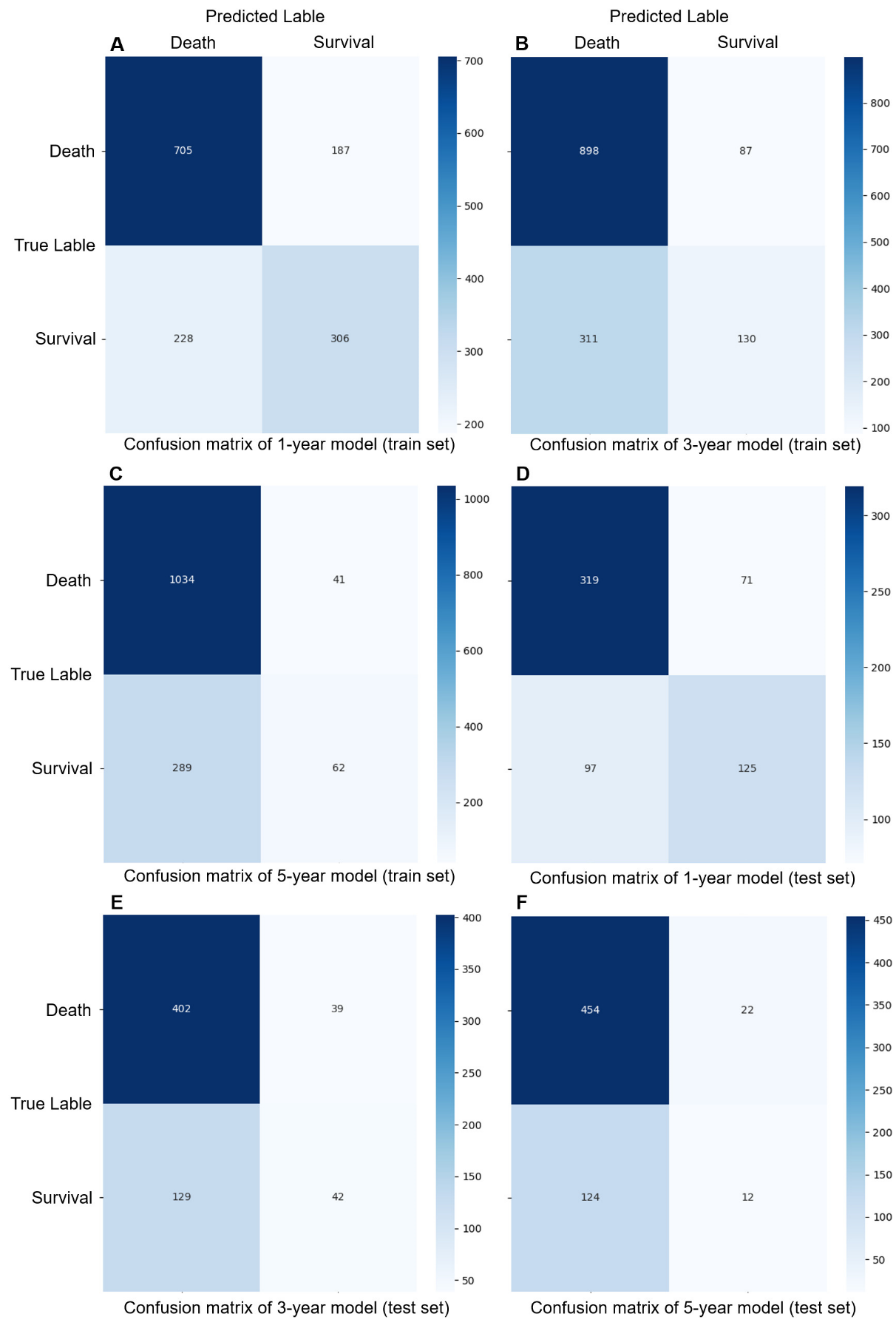
**

**Supplementary Fig.1** Confusion matrix of the XGBoost model’s predicted results in the training and test sets. Confusion matrices for **A** 1-year, **B** 3-year, and **C** 5-year prognostic models in the training set; and **D** 1-year, **E** 3-year, and **F** 5-year prognostic models in the test set. *XGBoost* extreme gradient boosting


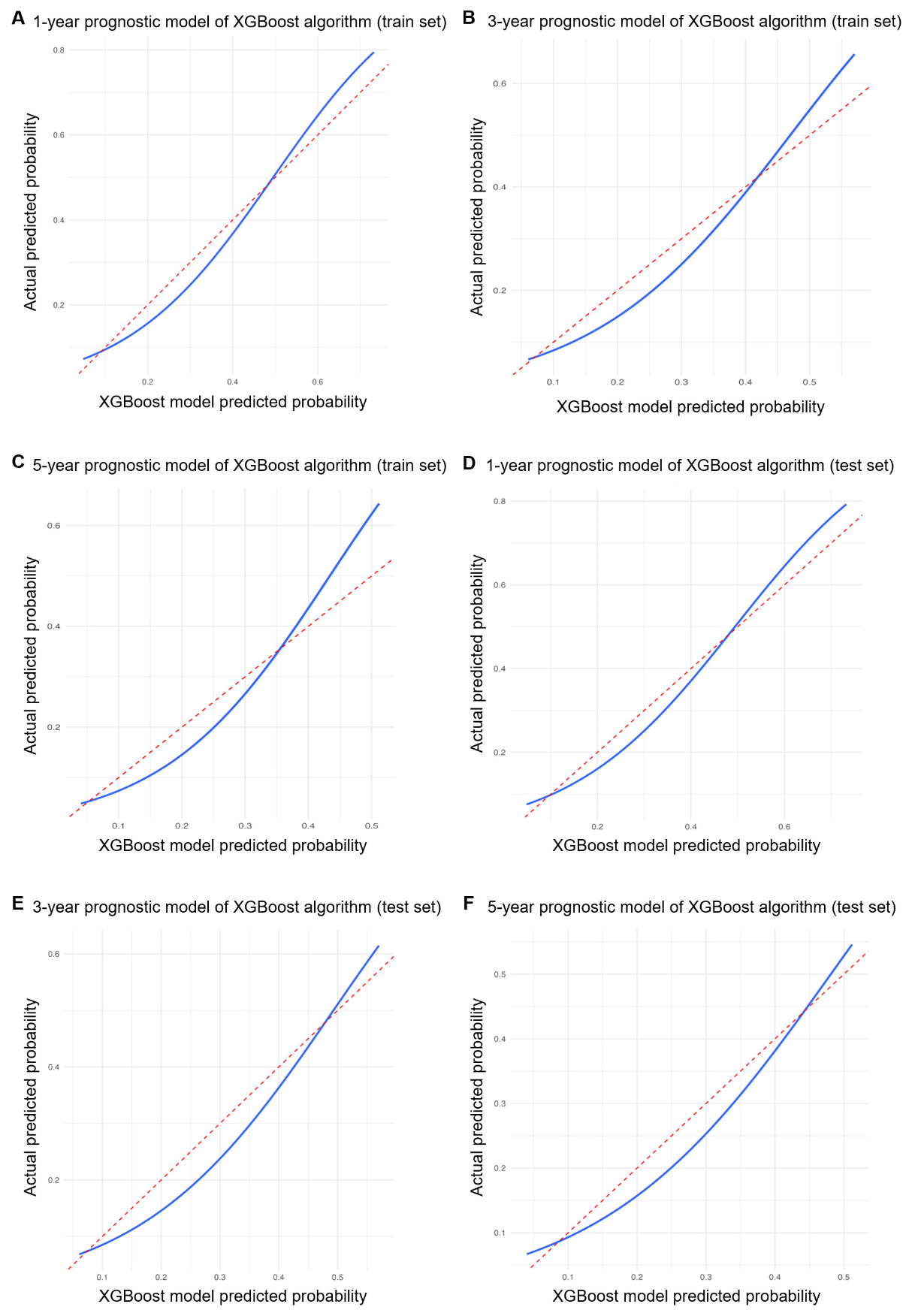


**Supplementary Fig.2** Calibration curves of the XGBoost model in the training and test sets. Calibration curves for **A** 1-year, **B** 3-year, and **C** 5-year prognostic models in the training set; and **D** 1-year, **E** 3-year, and **F** 5-year prognostic models in the test set. *XGBoost* extreme gradient boosting

**Supplementary Table 1**  Performance of prognostic models built by machine learning algorithms in the training and test sets (accuracy)

|  | **1-year survival** | **3-year survival** | **5-year survival** |
| --- | --- | --- | --- |
| Training set |  |  |  |
| XGBoost | 0.709 | 0.721 | 0.778 |
| LR | 0.701 | 0.710 | 0.762 |
| SVM | 0.626 | 0.691 | 0.754 |
| RF | 0.764 | 0.703 | 0.786 |
| KNN | 0.703 | 0.727 | 0.785 |
| ID3 | 0.699 | 0.721 | 0.768 |
| Test set |  |  |  |
| XGBoost | 0.726 | 0.726 | 0.784 |
| LR | 0.686 | 0.708 | 0.770 |
| SVM | 0.637 | 0.721 | 0.778 |
| RF | 0.717 | 0.722 | 0.779 |
| KNN | 0.627 | 0.686 | 0.740 |
| ID3 | 0.703 | 0.711 | 0.771 |

*ROC* receiver operating characteristic curve; *XGBoost* extreme gradient boosting; *LR* logistic regression; *SVM* support vector machine; *RF* random forest; *KNN* K-nearest neighbor; *ID3* decision tree
